# Clinical and genetic characteristics of a case of Koolen-De Vries syndrome caused by *KANSL1* gene mutation and literature review: A case report

**DOI:** 10.1097/MD.0000000000040923

**Published:** 2024-12-06

**Authors:** Haozheng Zhang, Limei Yuan, Meili Fan, Zhaotian Liu, Yuxi Yan, Qinghua Liu, Kaihui Zhang, Chunmiao Li, Deyao Liu

**Affiliations:** a Institute of Pediatric Research, Children’s Hospital Affiliated to Shandong University, Jinan, China; b Obstetrical Department, Jinan Fifth People’s Hospital, Jinan, China; c Enuresis Clinic of Tuina Department, Children’s Hospital Affiliated to Shandong University, Jinan, China; d Medical Imaging Department, Children’s Hospital Affiliated to Shandong University, Jinan, China; e Ultrasound Department, Children’s Hospital Affiliated to Shandong University, Jinan, China; f Blood Transfusion Department, Children’s Hospital Affiliated to Shandong University, Jinan, China; g Shandong Vocational College of Special Education, Jinan, China.

**Keywords:** genetic variation, *KANSL1* gene, Koolen-De Vries syndrome

## Abstract

**Rationale:**

Koolen-De Vries syndrome (KdVS, OMIM: 612452), also known as 17q21.31 microdeletion syndrome, is an autosomal dominant genetic disease. In the study, we analyze of clinical phenotype and gene variation of a child with Koolen-De Vries syndrome, review the literature to improve the understanding of the disease.

**Patient concerns:**

The patient is a male, aged 1 month and 3 days. The patient has poor airway development, difficulty weaning from respiratory support, seizures, and recurrent low granulocyte counts.

**Diagnoses:**

High-throughput sequencing showed a heterozygous mutation NM_001193466.1: c.1574_1578del (P.525HFS *24) in the *KANSL1* gene of the proband, which was considered a new mutation since neither of his parents carried this mutation based on Sanger sequencing results. Combining clinical features and genetic results, the proband was diagnosed as KdVS.

**Interventions and outcomes:**

The patient was in good condition after receiving bronchoscopy and laser interventional therapy, meeting the criteria for discharge. Follow-up for 1 year and 6 months indicated that the patient’s physical signs were normal and there was no recurrence.

**Lessons:**

According to literature review, KdVS is a multi-organ disease characterized by feeding difficulties, seizures, characteristic facial features, dysplasia of the respiratory system and cardiac abnormalities. In this study, laryngeal malacia accounted for 23.2% of the clinical manifestations of KdVS patients, limb convulsions/seizures accounted for 62.5%, and cardiac development defects accounted for 23.5%. The disease was rare in China and had a variety of clinical manifestations. The summary of reported cases can enable doctors to have more understanding of the disease. The new mutations enrich the *KANSL1* gene mutation spectrum.

## 1. Introduction

Koolen-de Vries syndrome (KdVS, OMIM: 612452), also known as chromosome 17q21.31 microdeletion syndrome, is an autosomal dominant genetic disorder triggered by one of the following 3 genetic variations: microdeletion of chromosome 17q21.31; heterozygotic mutation in *KANSL1* gene; and insufficient *KANSL1* haploid associated with chromosome rearrangement.^[1,2]^ With an incidence of about 1:16,000,^[2]^ KdVS involves with multiple systems and is characterized by neonatal hypotonia, feeding difficulties, developmental delays, speech disorders, seizures, characteristic facial deformities, musculoskeletal abnormalities, congenital heart defects, kidney and genitourinary system abnormalities, as well as ectodermal abnormalities.^[1,3,4]^

As an autosomal dominant inherited disorder, KdVS has proved to be caused by chromosome 17q21.31 deletion or pathogenic mutation of *KANSL1* gene in almost all the cases.^[4]^ In this study, whole exon high-throughput sequencing for high-risk neonates was conducted on a child with airway dysplasia and convulsive seizures resulted from unexplained intracranial hemorrhage, with the findings indicating heterozygous variation of *KANSL1* gene c.1574_1578del (p.P525Hfs*24) and a subsequent diagnose of KdVS. Considering that this genovariaton has never been reported in Human Gene Mutation Database, we particularly looked into the genetic etiology of this child, aiming to providing a theoretical basis for the diagnosis and genetic counseling of KdVS.

## 2. Objects and methodology

The proband is a male of 1 month and 3 days old whose mother underwent a cesarean section due to fetal distress, placenta praevia, vasa praevia, and velum attachment of the umbilical cord. He was admitted to ICU after birth in view of the manifestations such as dyspnea, positive three-concave sign, convulsion, and paroxysmal limb twitching. Afterwards, the child was transferred to our hospital owing to narrowing at the beginning of the laryngeal cavity and trachea. The physical examination revealed immature development, malnutrition, clear consciousness, normal reflexes in the nervous system, and slight shortness of breath. The chest examination showed a normal thorax and regular breathing rhythm, but with noticeably depressed suprasternal fossa during inhalation, positive three concave signs, and rough breathing sounds in both lungs. No other abnormalities were observed in the physical examination.

**Figure 1.**
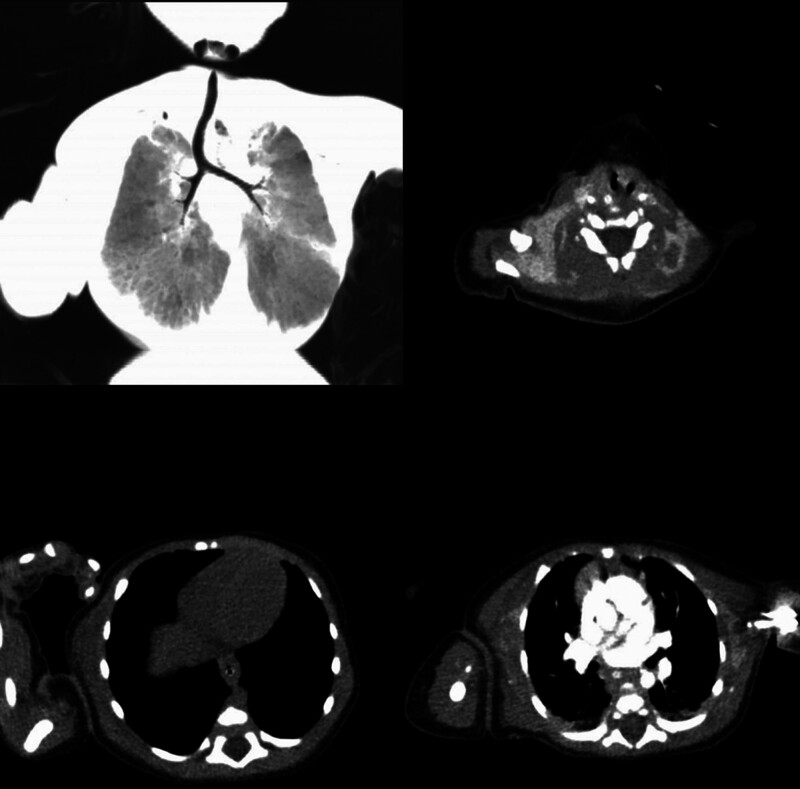
CT images of the child. The CT results show blurry images in part of the tissue structures and interstitial spaces of the neck and chest, along with soft tissue edema and air accumulation. The upper tracheal segment was tortuous and irregular. The above findings are consistent with pneumonia in accompany with partial pulmonary consolidation and air retention. Besides, multiple segmental bronchi are vaguely outlined, with narrowed bronchial lumen observed.

Chest CT: Blurry images were noted in some tissue structures and interstitial spaces of the neck and chest, accompanied by soft tissue edema and air accumulation. The upper tracheal segment was tortuous and irregular. These findings indicated pneumonia in accompany with partial pulmonary consolidation and air retention. Besides, multiple segmental bronchi were vaguely outlined, with narrowed bronchial lumen observed as shown in Figure 1.

Tracheobronchoscopy: Mild laryngeal chondromalacia (type I) was observed, along with concurrent pharyngeal cavity relaxation and laryngeal reflux. The examination also revealed changes of external pressure in the middle and lower tracheal section, stenosis in the opening of the right upper lobar bronchus, endotracheitis and endobronchitis, as well as cardiac relaxation.

Heart color ultrasound: multiple foramen atrial septal defect.

The patient was in good condition after receiving bronchoscopy and laser interventional therapy, meeting the criteria for discharge. Follow-up for one year and six months indicated that the patient’s physical signs were normal and there was no recurrence.

This study was approved by the Ethics Committee of Children’s Hospital affiliated to Shandong University (SDFE-IRB/*P*-2023011). Both parents have signed the informed consent.

### 2.1. Methods

#### 2.1.1. Genome extraction

In this study, 3 mL of peripheral blood were respectively collected from the child and his parents, with EDTA used for anticoagulation. Peripheral blood DNA was extracted using the blood genome extraction kit produced by Tiangen Biochemical Technology (Beijing, China) Co., LTD.

#### 2.1.2. High-throughput sequencing and Sanger sequencing

The DNA library was prepared by hybrid capture in the wake of segmentation, DNA splicing, amplification and purification of the children’s genomic DNA. Subsequently, the exons and peripheral intron regions (20 bp) of 20,099 genes in the human whole-exome were determined by high-throughput sequencing platform, followed by Sanger sequencing for validation of the detected pathogenic sites in the child and his parents.

#### 2.1.3. Pathogenicity of the mutations

The sequencing data were compared to the reference sequence of the human genome hg19 (GECh37) provided by UCSC using NextGene v2.3.4, followed by an evaluation on the coverage of the target area and the sequencing quality. The pathogenicity analysis of the variations detected in this study was performed according to the 2015 guideline from American College of Medical Genetics.

## 3. Results

### 3.1. High-throughput sequencing and Sanger sequencing

**Figure 2.**
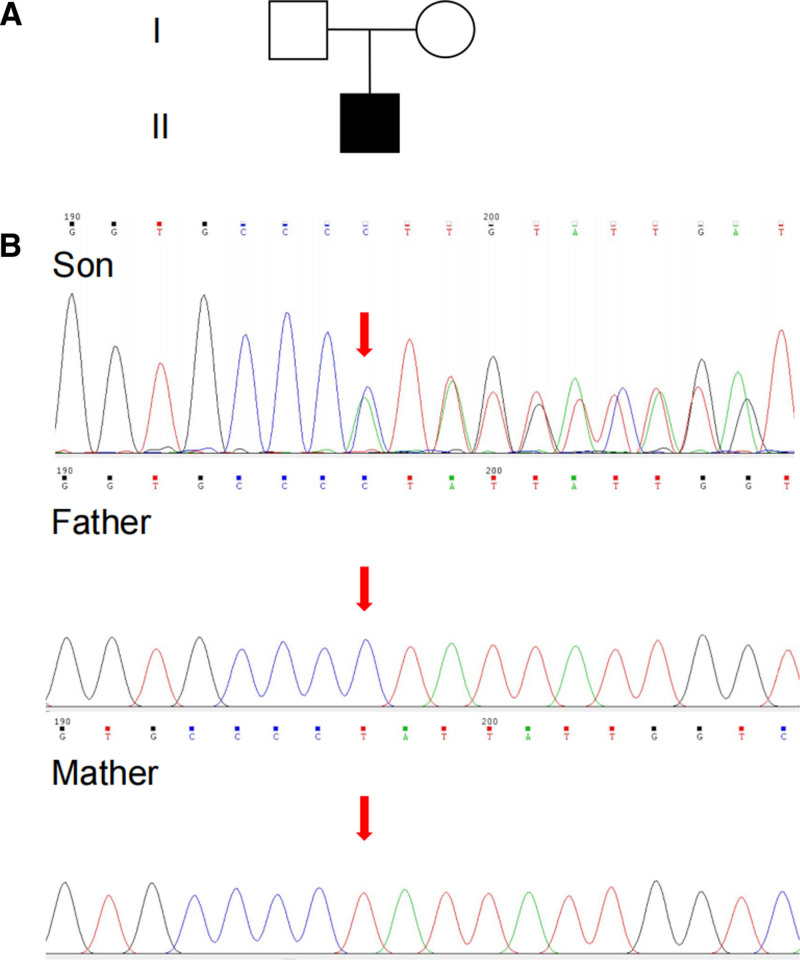
Family tree diagram and Sanger sequencing diagram. The mutation was verified by Sanger sequencing. There is a c.1574_1578del variant in exon 4 of the *KANSL1* gene on chromosome 17, which is a de novo variant that has never been discovered before.

High-throughput sequencing showed a heterozygous mutation NM_001193466.1: c.1574_1578del (P.525HFS *24) in the *KANSL1* gene of the proband, which was considered a new mutation since neither of his parents carried this mutation based on Sanger sequencing results, as shown in Figure 2.

### 3.2. Pathogenicity analysis of the variation sites

A heterozygous variant NM_001193466-1: c.1574_1578del (p.P525Hfs*24) was identified in *KANSL1* gene of the child, that is, the 1574-1578 bases in the coding region were missing. This mutation caused proline to be replaced by histidine as the 525th amino acid of the encoding protein, therefore ending the translation at the 24th amino acid and consequently, terminating the protein synthesis prematurely. Subsequently, Sanger sequencing confirmed that neither of the subject’s parents carried such a mutation, so it was likely to be a de novo variant or a parental germ cell-mediated chimera.

According to American College of Medical Genetics guidelines, the variant c.1574_1578del (p.P525Hfs*24) in *KANSL1* gene was determined to be a pathogenic mutation (PVS1 + PS2 + PM2). To be specific, PVS1, which has never been reported in Human Gene Mutation Database, occured on exon 4 (14 exons in total) of transcript NM_001193466.1. It may activate the nonsense-mediated mRNA decay and thus compromise the gene’s function of encoding protein products; PS2: This variant is a de novo variant (PS2); PM2: The variation was not recorded in the GnomAD database.

## 4. Discussion

As first reported in 2012, KdVS was primarily caused by a 500 to 650 kb microdeletion on chromosome 17q21.31. Studies have shown that the major clinical symptoms of the patients with pathogenic Single-nucleotide Variants on *KANSL1* gene are consistent with the manifestation of 17q21.31 microdeletion syndrome, which proves that insufficient haploid of *KANSL1* can result in a complete KdVS phenotype.^[1]^

Encoded by *KANSL1* gene, KAT8 regulatory nonspecific lethal complex Subunit 1 is composed of KANSL1, KANSL2, KANSL3, WDR5, MCRS1, PHF20, and histone acetyltransferase (KAT), and regulates global transcription by modifying histones.^[1,4]^ Deletion of 17q21.31 (hg19 chr17:g.43700000_44300000) involves with multiple genes such as CRHR1 (OMIM*122561), STH (OMIM*607067), MAPT (OMIM*157140), and KANSL1 (OMIM*612452).^[1,2]^ As KdVS is an autosomal dominant inherited disease, the majority of the affected patients are single cases in the family at the same time with a low recurrence rate (<1%). However, the incidence is still higher than that among the general population due to the possibility of parental germ cell chimerism.^[4,5]^

Koolen-de Vries syndrome is a rare genetic disease with a prevalence of about 1:16,000 in the general population. Since patients with this disease usually manifest multi-system and multi-organ dysplasia, the clinical phenotype varies greatly, thus leading to underdiagnosis in some cases.^[2]^ In the present study, a literature review was also performed based on this case and the other 184 patients retrieved from the literature. Specifically, “Koolen-de Vries syndrome” was used as the key words to search for the literature published before October 2022 in Chinese National Knowledge Infrastructure (CNKI), Wanfang database and pubmed database, with a total of 184 reports retrieved from 48 English and 1 Chinese literature. The reported cases were mainly distributed in Europe and the United States. According to statistics, the patients were characterized by multi-system and multi-organ developmental abnormalities as well as characteristic facial features (Table 1). Data analysis revealed low birth weight (10.0%) as well as growth and development disorders (22.2%) to be the major presentation in terms of growth parameters. The clinical manifestations mainly included feeding difficulties (49.0%), intellectual deficiency (77.4%), epilepsy (62.5%), friendly behavior (62.3%), language disorders (53.9%) and central nervous system abnormalities (46.3%). Moreover, the characteristic facial features were spherical nose (68.5%), long face (52.8%), as well as tubular and pear-shaped nose (40.6%).^[4–28]^

**Table 1:** List of clinical characteristic features

Clinical features	KdVS (n = 184)	
	n	%
Growth parameter		
Low birth weight	10	17/170
Short stature	22.2	38/171
Microcephaly	1.1	1/87
Macrocephaly		
Neurological features		
Hypotonia	26.8	37/138
Feeding difficulties	49.0	75/153
Mental deficiency	77.4	120/155
Mild	22.5	35/155
Moderate	32.2	50/155
Severe	23.2	36/155
Weak myoelectric activity	33.6	42/125
Language barrier	53.9	68/126
Seizures/abnormal EEG	62.5	105/168
Friendly behavior	62.3	73/117
Abnormal CNS structure	46.3	64/138
Ventricular enlargement	26.1	23/88
Neuropsychological disorder	28.1	27/96
Physiological defects		
Long face	52.84	65/123
Eyelid lift	31.3	30/96
Narrow/short eyelid cleft	22.9	22/96
Upper eyelid ptosis	31.7	29/123
Epicanthal fold	26.6	33/124
Tubular or pear-shaped nose	40.6	39/95
Spherical nose	68.5	74/108
Lower lip valgus	36.3	40/110
Big ears/protruding ears	25.2	30/119
Musculoskeletal abnormalities		
Tracheal/laryngeal malacia	23.2	29/125
Scoliosis/kyphosis	33.0	40/121
Hyperrelaxation of joints	29.7	28/94
Foot deformity	27.3	32/117
Visual and hearing impairments		
Farsightedness	20.1	23/114
Squint	29.2	43/147
Hearing impairment	17.3	25/144
Heart defects	23.5	28/119
Abnormal genitourinary system		
Cryptorchidism	26.5	22/83
Ectodermal abnormalities		
Polypigmented nevus	14.2	17/119

**Figure 3.**
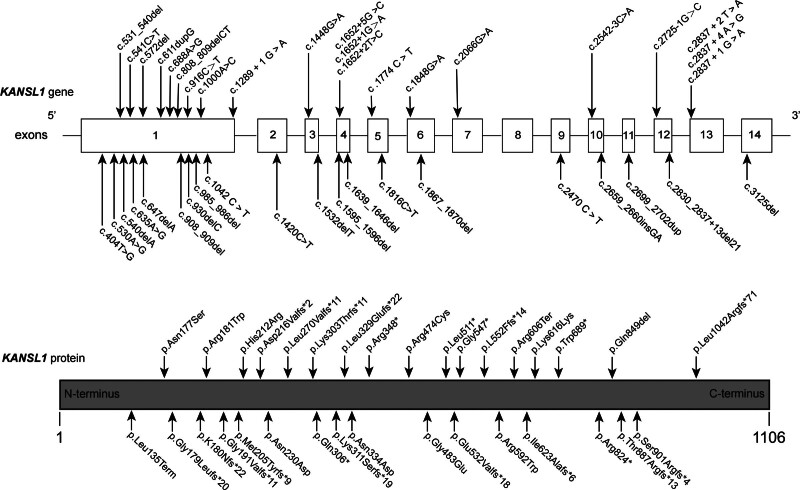
Schematic Diagram of Pathogenic Mutations in the KANSL1 Gene. Schematic diagram of all reported pathogenic mutations of the KANSL1 gene in literature searches.

Among the 184 patients reviewed in the literature, 41 cases were caused by heterozygous pathogenic mutations of *KANSL1* gene (Table 2), and 143 cases were attributed to microdeletions in chromosome 17q21.31. Through literature review, we found that a total of 41 *KANSL1* gene variants were thoroughly reported (Table 2), including frameshift variant in 16 cases (39.0%), missense variant in 11 cases (26.8%), nonsense variant in 4 cases (9.8%), splice site variant in 9 cases (22.0%), and truncating variant in 1 case (2.4%).^[1,7,8,12,14,17,22,23,25,29–32]^ The diagram of gene variation is shown in Figure 3.

**Table 2:** List of KANSL1 gene mutations

Proband	Nucleotide alteration	Variant type	Coding sequence alteration	Literature
1	c.916C>T	Nonsense variant	p.(Gln306*)	[1]
2	c.1652 + 1G>A	Splice site variant	p.(?)	[1]
3	c.985_986del	Truncating frameshift variant	p.(Leu329Glufs*22)	[1]
4	c.1867_1870del	Truncating frameshift variant	p.(Ile623Alafs*6)	[1]
5	c.531_540del	Truncating frameshift variant	p.(Gly179Leufs*20)	[1]
6	c.2725-1G>C	Splice site variant	p.(?)	[1]
7	c.1639_1646del	Truncating frameshift variant	p.(Gly547*)	[1]
8	c.572del	Truncating frameshift variant	p.(Gly191Valfs*11)	[1]
9	c.2699_2702dup	Truncating frameshift variant	p.(Ser901Argfs*4)	[1]
10	c.3125del	Truncating frameshift variant	p.(Leu1042Argfs*71)	[1]
11	c.908_909del	Truncating frameshift variant	p.(Lys303Thrfs*11)	[1]
12	c.1042 C > T	Nonsense variant	p.Arg348*	[7]
13	c.2470 C > T	Nonsense variant	p.Arg824*	[7]
14	c.2066G > A	Nonsense variant	p.Trp689*	[8]
15	c.808_809delCT	Truncating frameshift variant	p.Leu270Valfs*11	[8]
16	c.2542-3C > A	Splice site variant	p.(p.Gln849del)	[12]
17	c.1448G > A	Missense variant	p.(Gly483Glu)	[12]
18	c.530A > G	Missense variant	p.(Asn177Ser)	[12]
19	c.1774 C > T	Missense variant	p.Arg592Trp	[14]
20	c.1595_1596del	Truncating frameshift variant	p.Glu532Valfs*18	[17]
21	c.541C > T	Missense variant	p.Arg181Trp	[22]
22	c.635A > G	Missense variant	p.His212Arg	[22]
23	c.688A > G	Missense variant	p.Asn230Asp	[22]
24	c.1000A > C	Missense variant	p.Asn334Asp	[22]
25	c.1816C > T	Missense variant	p.Arg606Ter	[23]
26	c.1652 + 2T > C	Truncating frameshift variant	p.L552Ffs*14	[23]
27	c.540delA	Truncating frameshift variant	p.K180Nfs*22	[25]
28	c.2659_2660insGA	Truncating frameshift variant	p.Thr887Argfs*13	[29]
29	c.2830_2837 + 13del21	Splice site variant	p.(?)	[29]
30	c.1532delT	Truncating variant	p.Leu511*	[29]
31	c.2837 + 1 G > A	Splice site variant	p.(?)	[29]
32	c.930delC	Truncating frameshift variant	p.Lys311Serfs*19	[29]
33	c.1289 + 1 G > A	Splice site variant	p.(?)	[29]
34	c.2837 + 2 T > A	Splice site variant	p.(?)	[29]
35	c.2837 + 4 A > G	Splice site variant	p.(?)	[29]
36	c.647delA	Truncating frameshift variant	p.Asp216Valfs*2	[29]
37	c.611dupG	Truncating frameshift variant	p.Met205Tyrfs*9	[29]
38	c.1652 + 5 G > C	Splice site variant	p.(?)	[29]
39	c.1848G > A	Missense variant	p.Lys616Lys	[30]
40	c.404T > G	Missense variant	p.Leu135Term	[31]
41	c.1420C > T	Missense variant	p.Arg474Cys	[32]

In the present study, the proband shares a high similarity with those previously reported cases from the perspective of the clinical manifestations. Meanwhile, a heterozygous mutation NM_001193466.1: c.1574_1578del (p.P525Hfs*24) was spotted in *KANSL1* gene, that is, the deletion of the 1574 to 1578 bases in the coding region, which leads to the replacement of proline by histidine in the 525th amino acid in the encoding protein and consequently, ending of the translation at the 24th amino acid as well as the premature termination of protein synthesis. Eventually the variation was identified as a pathogenic mutation. With the development of high-throughput sequencing technology, it will be more and more widely applied in the diagnosis of rare genetic diseases.

Nowadays, prenatal diagnostic techniques are universally used to prevent KdVS. For instance, Central Nervous System abnormalities detected in prenatal examinations during the third trimester of pregnancy, especially ventricular enlargement, should also be considered as prenatal ultrasound markers for this syndrome.^[3]^ In this case, the variation is diagnosed as a de novo mutation but without ruling out the possibility of germ cell chimerism. Therefore, prenatal diagnosis is also required in case of rebirth. In this study, new generation of sequencing technology was employed, with the results revealing for the first time that this pathogenic mutation was caused by base deletion in *KANSL1* gene. This has not been previously reported and thus has substantial significance for the study of the phenotype and genotype as well as the treatment of KdVS. In addition, next-generation sequencing technology also provides technical support for the early diagnosis of other rare genetic diseases.

## Author contributions

**Conceptualization:** Haozheng Zhang.

**Data curation:** Haozheng Zhang, Limei Yuan, Meili Fan, Zhaotian Liu, Yuxi Yan, Qinghua Liu, Kaihui Zhang, Chunmiao Li.

**Funding acquisition:** Haozheng Zhang, Meili Fan.

**Investigation:** Haozheng Zhang, Limei Yuan.

**Methodology:** Haozheng Zhang, Deyao Liu.

**Writing – original draft:** Haozheng Zhang, Limei Yuan, Deyao Liu.

**Writing – review & editing:** Haozheng Zhang, Meili Fan, Deyao Liu.
